# Hospital-acquired and zoonotic bacteria from a veterinary hospital and their associated antimicrobial-susceptibility profiles: A systematic review

**DOI:** 10.3389/fvets.2022.1087052

**Published:** 2023-01-09

**Authors:** Dikeledi C. Sebola, James W. Oguttu, Marleen M. Kock, Daniel N. Qekwana

**Affiliations:** ^1^Section Veterinary Public Health, Department of Paraclinical Sciences, Faculty of Veterinary Science, University of Pretoria, Pretoria, South Africa; ^2^Department of Agriculture and Animal Health, College of Agriculture and Environmental Sciences, University of South Africa, Johannesburg, South Africa; ^3^Department of Medical Microbiology, University of Pretoria, Pretoria, South Africa; ^4^Tshwane Academic Division, National Health Laboratory Service, Pretoria, South Africa

**Keywords:** hospital acquired infections (HAIs), zoonosis, veterinary, antimicrobial resistance (AMR), nosocomial, antimicrobials, multi-drug resistance

## Abstract

**Background:**

Hospital-acquired infections (HAIs) are associated with increased mortality, morbidity, and an economic burden due to costs associated with extended hospital stays. Furthermore, most pathogens associated with HAIs in veterinary medicine are zoonotic. This study used published data to identify organisms associated with HAIs and zoonosis in veterinary medicine. Furthermore, the study also investigated the antimicrobial-susceptibility profile of these bacterial organisms.

**Methods:**

A systematic literature review was conducted in accordance with the Preferred Reporting Items for Systematic Reviews and Meta-analyses (PRISMA) guidelines. Search terms and five electronic databases were used to identify studies published over 20 years (2000–2020). The risk of bias was assessed using the “Strengthening the Reporting of Observational Studies in Epidemiology-Vet” (STROBE-Vet) checklist.

**Results:**

Out of the identified 628 papers, 27 met the inclusion criteria for this study. Most studies (63%, 17/27) included were either from small animal or companion animal clinics/hospitals, while 5% (4/27) were from large animal clinics/hospitals inclusive of bovine and equine hospitals. Hospital-acquired bacteria were reported from environmental surfaces (33%, 9/27), animal clinical cases (29.6%, 8/27), and fomites such as cell phones, clippers, stethoscopes, and computers (14.8%, 4/27). *Staphylococcus* spp. was the most (63%; 17/27) reported organism, followed by *Escherichia coli* (19%; 5/27), *Enterococcus* spp. (15%, 4/27), *Salmonella* spp. (15%; 4/27), *Acinetobacter baumannii* (15%, 4/27), *Clostridioides difficile* (4%, 1/27), and *Pseudomonas aeruginosa* (4%; 1/27). Multidrug-resistant (MDR) organisms were reported in 71% (12/17) of studies linked to Methicillin-resistant *Staphylococcus aureus* (MRSA), Methicillin-resistant *Staphylococcus pseudintermedius* (MRSP), *Enterococcus* spp., *Salmonella* Typhimurium*, A. baumannii*, and *E. coli*. The *mec*A gene was identified in both MRSA and MRSP, the *bla*CMY-2 gene in *E. coli* and *Salmonella* spp., and the *van*A gene in *E. faecium* isolate. Six studies reported organisms from animals with similar clonal lineage to those reported in human isolates.

**Conclusion:**

Organisms associated with hospital-acquired infections and zoonosis have been reported from clinical cases, environmental surfaces, and items used during patient treatment and care. *Staphylococcus* species is the most reported organism in cases of HAIs and some isolates shared similar clonal lineage to those reported in humans. Some organisms associated with HAIs exhibit a high level of resistance and contain genes associated with antibiotic resistance.

## 1. Introduction

Hospital-acquired infections (HAIs) in both veterinary and human medicine are associated with increased mortality, and morbidity, and are an economic burden due to the increased cost of extended hospital stay and treatment options (1, 2). The most reported HAIs include surgical wounds, urinary tract, and gastrointestinal infections (1–3) and are often associated with bacteria such as *Enterococcus* species (spp.), *Escherichia coli, Staphylococcus* spp., *Enterobacter* spp., *Klebsiella* spp., *Acinetobacter* spp., and *Pseudomonas* spp. (3–6).

Available evidence suggests that HAIs associated with *Enterococcus* spp., *Escherichia coli, K. pneumoniae*, and *S. aureus* are on the increase in veterinary medicine (7–10). There are also reports of vancomycin-resistant enterococci (VRE), multidrug-resistant (MDR) *E. coli*, carbapenem-resistant *A. baumannii*, carbapenem-resistant *P. aeruginosa*, carbapenem-resistant and extended-spectrum β-lactamase (ESBL) producing *Enterobacteriaceae* (3–6, 11–13), with limited treatment options and poor prognosis (1, 5, 6, 11, 13). It is estimated that 60% of emerging infectious diseases are likely to come from animals (14, 15). Of concern is that bacteria associated with HAIs in veterinary settings could be contributing to the emergence of these new diseases (6, 16). Since the veterinary hospital environment is a human-animal interface, it remains a potential source of zoonotic pathogens (6, 17). Therefore, veterinary healthcare workers and animal owners are at an increased risk of contracting various zoonotic infections (14, 15). This is likely to put financial stress on the human health system especially in developing countries (18). In view of this, continuous surveillance of hospital-acquired and zoonotic pathogens in veterinary medicine should be done to better quantify the risk of transmission to personnel and animal owners (19, 20).

Systematic review studies have suggested that improving surveillance systems is critical in the prevention of HAIs and in reducing the emergence of antimicrobial-resistant pathogens (6, 21). Therefore, a holistic approach is needed to investigate the types of disease agents, hosts, the antimicrobial-resistance profile of the organism, and the virulence of the organisms associated with HAIs in veterinary medicine (17).

This study describes the occurrence and antimicrobial-susceptibility profiles of bacterial organisms associated with HAIs and zoonosis in veterinary medicine. It addresses the following research questions: (1) Which bacteria associated with HAIs and zoonotic diseases have been reported in veterinary hospitals? (2) What are the antimicrobial resistance profiles of these bacteria?

## 2. Materials and methods

The systematic literature review was conducted using the Preferred Reporting Items for Systematic Reviews and Meta-analyses (PRISMA) guidelines (22). Keywords and synonyms used in various databases included hospital-acquired organism or infection, nosocomial organism or infection, animal to animal infections, zoonotic infection, zoonosis, animal to human infections, veterinary hospital, and veterinary clinic.

### 2.1. Information source

Search terms and electronic databases used in this study are provided in Table 1. Since each database has a different search function, alternate search terms appropriate for each database were used. Boolean operators were utilized in all searches. A data search was conducted between June 2020 and December 2020. A follow-up search was performed in January 2021, however, there were no additional studies considered based on the inclusion criteria. Mendeley reference manager was used to store all studies and documents retrieved.

**Table 1:** Search terms and databases utilized to search for articles included in this review about hospital-acquired and/or zoonotic infections in veterinary facilities between 2000 and 2020.

**Publications**	**Search terms**
Science Direct	Veterinary AND “Infection Control” AND “hospital acquired infection OR nosocomial” AND zoonoses OR zoonotic OR zoonosis
	“Veterinary hospital OR clinic” AND “hospital acquired infections” OR nosocomial AND zoonoses OR zoonotic OR zoonosis
	“Systematic literature review” AND “Hospital acquired infection OR nosocomial” AND “zoonoses OR zoonosis OR zoonotic” AND veterinary
	“Hospital acquired infection OR nosocomial” AND “zoonoses OR zoonosis OR zoonotic” AND veterinary
	Veterinary AND “hospital acquired infection OR nosocomial”
	“Veterinary hospital” AND “hospital acquired infection OR nosocomial” NOT “Human hospital”
PubMed	“Hospital acquired infections OR nosocomial” AND veterinary AND “zoonosis or zoonoses or zoonotic”
	“Infection prevention and control” [All Fields] AND veterinary AND “hospital acquired infection or nosocomial” AND zoonoses
	“Hospital acquired infections OR nosocomial” AND veterinary
Web of Science	“Hospital acquired infections” AND veterinary
	“Hospital acquired infections” AND “veterinary hospital”
	“Hospital acquired infections” AND “zoonotic infections” AND “Veterinary hospital”
Google Scholar	“Systematic literature review” AND “Hospital acquired infection OR nosocomial” AND “zoonoses OR zoonosis OR zoonotic” AND veterinary
	“Hospital acquired infection OR nosocomial” AND “zoonoses OR zoonosis OR zoonotic” AND veterinary
	“Hospital acquired infection OR nosocomial” AND “veterinary hospital”
Scopus	“Hospital acquired infection” AND zoonoses AND veterinary
	Nosocomial AND zoonoses AND veterinary

### 2.2. Eligibility criteria

Only manuscripts published in peer-reviewed journals were considered for inclusion in this study. Primary research articles written in English and published between 2000 and 2020 were selected. The microbiological data included bacterial isolates from HAI cases, hospital environmental screening, fomites from veterinary hospitals, and zoonotic cases in veterinary hospitals. In addition, the antimicrobial resistance profiles of the different bacteria were also extracted. The inclusion and exclusion criteria are listed in Table 2. Two investigators (DC, DN) independently screened the titles and abstracts from the searches. Any disagreements were settled by discussion. The use of either the CLSI or EUCAST guidelines was not considered an eligibility criterion in this study since some studies report potential discrepancies between the results of the antimicrobial resistance based on CLSI and EUCAST (23), while others report comparable antibiotic susceptibility rates between CLSI and EUCAST breakpoints (24, 25).

**Table 2:** Inclusion and exclusion criteria of articles reporting on hospital-acquired and/or zoonotic infections in veterinary facilities between 2000 and 2020.

**Inclusion criteria**	**Exclusion criteria**
Veterinary medicine studies	Human hospital studies
Small animal/Companion animal Equine/Large animals	Farms, home studies
Peer-reviewed research	Reviews
Year 2000–2020	Policies, Government documents and conference reports, Book chapters
Studies in English	Non-English studies
Infection prevention and control practices	
(Environmental screening)	

### 2.3. Study selection and data items

For each study that met the selection criteria for inclusion, the following data were extracted: author, year, the theme of study (HAIs or zoonotic studies), and the antimicrobial resistance profile.

## 3. Results

### 3.1. Study selection

**Figure 1:**
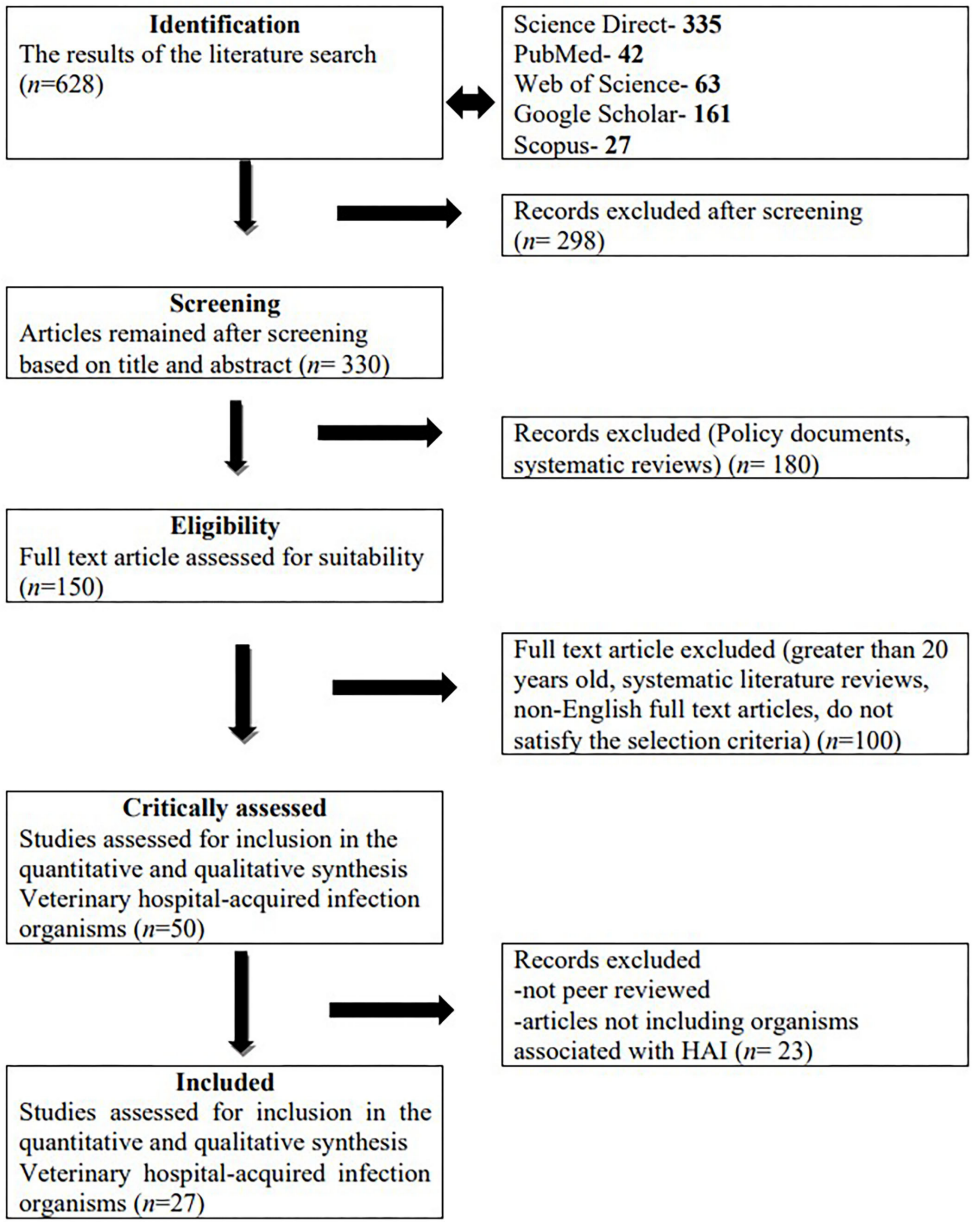
Summary of study selection and exclusion using the preferred reporting items for systematic reviews and meta-analyses (PRISMA) guidelines.

Initially a total of 628 studies were identified. After initial screening, 330 articles remained. Based on the eligibility screening criteria, 48 studies remained and were further critically assessed. A total of 27 studies met the inclusion criteria and were further analyzed (Figure 1).

### 3.2. Risk of bias

Strengthening the Reporting of Observational Studies in Epidemiology (STROBE-Vet) statement is a 22-item tool that allows a systematic way of reporting on veterinary observational studies. The STROBE statement was developed to guide the reporting of observational studies related to human health. These methods have been adopted and used for standardized reporting guidelines for observational studies in veterinary medicine (21, 26). Identified studies that met the inclusion criteria were cross-sectional and cohort studies (26). Each study was assessed individually according to each of the 22 items.

Items were considered to have been reported sufficiently if the studies provided a detailed abstract and clear title (item one), background, and rationale (item two), stated the objectives (item three), presented key elements of the study design (item four), described the sample size (item 11), reported outcomes for the study (items 14 and 15), provided estimates and parameters (item 16), summarized key results regarding study objectives (item 18 and 19), interpreted results (item 20), discussed the results (item 21), and stated the funding source as well as the role of authors as described by Sergeant et al. (26).

Only two studies (7%, 2/27) reported on all STROBE-Vet items (27, 28). Based on STROBE-Vet, item 1 was partially attained by 19/27 (70%) studies as they excluded the study design and was fully attained by 8 (29%) studies. Items 6, 13,14, and 20 were fully attained by all the studies, Items 2, 4, 5, and 16 were fully attained by 26 (96%) of the studies, items 3,15,17, and were fully attained by 25 (93%) of the studies, item 7 and 18 were fully attained by 24 (89%) of the studies, items 9 and 19 were fully attained by 21 (78%) of the studies, items 11 and 21 were fully attained by 20 (74%) studies and item 10 was fully attained by 63% of the studies. Twelve (12; 44%) studies provided the funding sources, twelve (12; 44%) studies declared no conflict of interest, three studies (3; 11%) mentioned the contribution of each author, and three (3, 11%) provided ethical clearance declarations (Annexure A).

### 3.3. Sources of data

All the studies reviewed were observational. More than half (18; 67%) of the reported studies were cross-sectional studies, three (11%) were case-controlled studies (reported following an outbreak), and six (22%) were retrospective studies.

Twenty-four (89%) studies focused on a specific bacterium, whereas the other three studies (11%) (17, 29, 30) reported generally on the bacteria associated with HAIs. Most studies (78%) (9, 19, 20, 30–47) investigated the occurrence of HAIs in a single facility, five (19%) (3, 17, 27–29) studies investigated multiple facilities in an area, and one (4%) (48) study did not specify the area of study.

Seventeen (17/27, 63%) studies were from either the small animal or companion animal clinics/hospitals (3, 9, 17, 20, 27–33, 37, 41–44, 48). followed by both bovine (38, 42, 49, 50) (4/27, 15%) and equine medicine (19, 46, 47). Three (3/27, 11%) studies were a combination of small animals, large animals, and poultry (35, 36, 40). One (1/27, 4 %) study did not identify the type of veterinary clinic or hospital (39).

Within the hospital settings, bacteria associated with HAIs were reported from environmental surfaces (9/27; 33%) (9, 17, 20, 36, 39, 40, 42, 44, 47), animal cases (8/27; 30%) (3, 19, 28, 31, 33, 35, 43, 47), and commonly used fomites such as clothing, cell phones, clippers, stethoscopes, and computers (4/27, 15%) (29, 32, 36, 41). Only three studies (3/27, 11%) isolated bacteria from humans who have regular contact with animals (17, 32, 38).

The antimicrobial resistance profile of the different organisms was provided in eighteen (17/27, 63%) studies (3, 9, 17, 20, 28, 31, 33–37, 39, 40, 42, 43, 46, 47), while nine (9/27, 33%) studies did not report on the antimicrobial resistance patterns (19, 27, 29, 30, 32, 38, 41, 44, 48). Thirteen studies (13/27, 48%) further characterized the microorganisms using pulsed-field gel electrophoresis (PFGE) and polymerase chain reaction (PCR) assays (3, 19, 20, 28, 31, 33–35, 37, 40, 45–47).

### 3.4. Bacterial species associated with hospital-acquired infections

*Staphylococcus* spp. were the most (17/27, 63%) reported pathogens associated with HAIs, followed by *Escherichia coli* (5/27; 19%), *Enterococcus* spp. (4/27; 15%)*, Salmonella* spp. (4/27; 15%), *A. baumannii* (4/27; 15%), *C. difficile* (1/27; 4%), and *P. aeruginosa*. (1/27; 4%). *Enterococcus faecalis* (3/4; 75%) and *E. faecium* (3/4; 75%) were the most reported among the *Enterococcus* species.

Among the *Staphylococcus* spp., 11 (11/17, 65%) were MRSA and six (6/17, 35%) were methicillin-resistant *S. pseudintermedius* (MRSP). Three out of five (3/5; 60%) studies reported MDR *Escherichia coli* isolates and one (1/5; 20%) study reported an extended spectrum β-lactamase (ESBL) producing *E. coli*. Meanwhile, vancomycin-resistant *enterococci* were reported in one (1/4; 25%) study. *Salmonella* Typhimurium was reported as the common serotype in two of the four (2/4; 50%) studies. The other two of the four (2/4; 50%) studies reported the presence of MDR *Salmonella* (Table 3).

**Table 3:** Organism reported in hospital-acquired and/or zoonotic infections in veterinary facilities between 2000 and 2020.

**Bacteria**	**Citation**
*Staphylococcus* spp.	(3, 9, 17, 19, 20, 27, 29, 30, 32–34, 36, 37, 40–42, 44)
Methicillin-resistant *S. aureus*	(17, 20, 27, 32, 35–38, 40, 41, 51)
Methicillin-resistant *S. pseudintermedius*	(9, 17, 27, 32, 41, 44)
*Clostridium difficile*	(17)
*Enterococcus* spp.	(3, 17, 42, 43)
*E. faecalis*	(3, 42, 43)
*E. faecium*	(3, 42, 43)
Vancomycin-resistant enterococci	(17)
*Acinetobacter baumannii*	(3, 28, 29, 39)
*Escherichia coli*	(17, 29, 31, 39, 50)
Extended spectrum β-lactamase (ESBL)	(17)
Multidrug resistance *E. coli*	(17, 31, 39)
*Salmonella* spp.	(17, 38, 46, 47)
Multidrug-resistant *Salmonella*	(46, 47)
*Pseudomonas aeruginosa*	(42)

### 3.5. Sources of organisms associated with hospital-acquired infections

The following pathogens were detected in the hospital environmental surfaces, namely MRSA (17, 20, 37, 40), MRSP (42, 44), ESBL-producing *E. coli* isolates (17), VRE (17), *A. baumannii* (39), *C. difficile* (17) and *P. aeruginosa* (42). Common pathogens identified from hospital fomites included: MRSA (19, 32, 36, 41), MRSP (9, 17, 41), *Enterococcus faecalis* (42), and *A. baumannii* (29, 32, 39).

Among patients in hospital settings, MRSA was isolated from companions (35) and equine animals (19, 34). Multidrug resistant *Escherichia coli* was isolated from companion and bovine animals (31, 50). Additionally, *Enterococcus faecium, Enterococcus faecalis* (3, 46) and *A. baumannii* (3) were isolated from companion animals. *Salmonella* species were also isolated from patients (38, 47), healthy animals (46), and the hospital environment (17, 46) (Table 4).

**Table 4:** Sources of hospital acquired organisms based on the systematic reviewed papers published from 2000 to 2020.

**Source**	**^a^MRSA**	**^b^MRSP**	**^c^ESBL E. coli**	**^d^MDR** ***Escherichia coli***	***Enterococcus faecalis***	***Enterococcus faecium***	***C. difficile***	***P. aeruginosa***	***A. baumannii***	***Salmonella* spp**.
**Animal**										
Patients	(35) (34) (19)			(31) (50)	(3) (43)	(3) (43)			(3)	(47) (38)
Healthy										(46)
**Environment**										
Hospital	(17) (20) (40) (37)	(42) (44)	(17)				(17)	(42)	(39)	(17) (46)
Equipment	(19) (36) (32) (41)	(17) (9) (41)			(42)				(29) (32) (39)	
Healthcare workers	(42) (52) (27)	(42) (32)				(42)				
Pet Owners	(27)									
	(27, 34)									

^a^MRSA, Methicillin-resistant *Staphylococcus aureus*.

^b^MRSP, Methicillin-resistant *Staphylococcus pseudintermediu*s.

^c^ESBL-*E. coli*, Extended-spectrum beta-lactamase producing- *E. coli*.

^d^MDR-*E. coli*, Multidrug-resistant *E. coli*.

The healthcare workers (HCWs) harbored MRSA (27, 37, 42), MRSP (32, 42), *E. faecium* (42) and two studies reported MRSA among pet owners (27, 34). In addition, van Duijkeren et al. (35) and Hoet et al. (20) reported on the zoonotic potential of MRSA with van Duijkeren (35) identifying MRSA clusters in animals with a similar clonal lineage to that reported in humans (**Table 6**).

### 3.6. Antimicrobial resistance patterns of bacteria associated with hospital acquired infections

#### 3.6.1. Phenotypic resistance

Out of the 27 studies reviewed, 17 (63%) conducted an antimicrobial susceptibility test on the isolates. Among these, 12 (71%) studies reported isolates resistant to more than one antimicrobial. Bacteria resistant to multiple drugs identified included MRSA (20, 34, 40, 45), MRSP (42), *A. baumannii* (28, 39), *E. coli* (31, 48), *Salmonella* Typhimurium (46, 47), *E. faecalis* and *E. faecium* (43).

Methicillin-resistant *Staphylococcus aureus* isolates showed resistance toward ampicillin, amoxicillin, oxacillin, clindamycin, gentamycin, ciprofloxacin, cephalexin, enrofloxacin, cefuroxime, chloramphenicol, erythromycin, and kanamycin while MRSP isolates showed resistance toward azithromycin, oxacillin, penicillin, clindamycin, gentamycin, tetracycline, and ciprofloxacin. *Clostridioides difficile* showed resistance toward rifampin, moxifloxacin, and chloramphenicol. *Enterococcus faecalis* and *E. faecium* showed resistance toward ampicillin, tetracycline, ciprofloxacin, enrofloxacin, erythromycin, and rifampicin (43). *Enterococcus faecium* was also reported to be resistant to amoxicillin and vancomycin (42).

*Acinetobacter baumannii* exhibited resistance to amoxicillin, tetracycline (39), ciprofloxacin (28) and imipenem (28). While *E. coli* showed resistance to ampicillin, cefoxitin, oxacillin, and penicillin (31, 48) and *Salmonella* was resistant to ampicillin, amoxicillin, cefoxitin, gentamycin, tetracycline, chloramphenicol, rifampicin, and streptomycin (47) (Table 5).

**Table 5:** Phenotypic antimicrobial resistance profile of hospital-acquired infection organisms based on the systematically reviewed papers published from 2000 to 2020.

**Pathogens**	**AMP**	**AMX**	**CEF**	**AZI**	**OXA**	**PEN**	**CLI**	**GEN**	**TET**	**CIP **	**VAN**	**CFL**	**ENF**	**CFR**	**CHL**	**ERY**	**KAN**	**RIF**	**MOX**	**CLO**	**CPH**	**IMI**	**STR**	**AMX-C**
**Gram-positive bacteria**																								
^a^MRSA	(35)^*^ (20) (40) (43)	(37) (35) (20) (40)	(38)	(42)	(42) (20) (40)	(42) (40)	(37) (20) (42)	(35, 42) (20)		(20) (37)		(35)	(20) (35)	(37)	(37)	(37) (35) (20) (46)	(37)							
^b^MRSP				(42)	(42)	(42)	(42)	(42)	(42)	(42)														
*E. faecium*	(43) (42)	(42)						(40)	(42) (43)	(42) (43)	(42)		(43)					(43)						
*E. faecalis*	(43)							(40)	(43)	(43)			(43)			(43)		(43)						
*C. difficile*																		(19)	(19)	(19)				
**Gram-negative bacteria**																								
*E. coli*	(48) (31)		(48) (31)		(48)	(48)																		(50) (33)
*A. baumannii*		(39)							(39)	(28)		(28)	(28)									(19)		(28)
*Salmonella spp*.	(46) (47)	(46)	(46)					(46) (47)	(46) (47)						(46) (47)			(46)			(46)		(46) (43)	(46) (47)

AMP, Ampicillin; AMX, Amoxicillin; CEF, Cefoxitin; AMX-C, Amoxycillin-Clavulanic Acid; AZI, Azithromycin; OXA, Oxacillin; PEN, Penicillin; CLI, Clindamycin; GEN, Gentamicin; TET, Tetracycline; CIP, Ciprofloxacin; VAN, Vancomycin; LIN, Linezolid; CFL, Cephalexin; ENF, Enrofloxacin; CFR, Cefuroxime; CHL, Chloramphenicol; ERY, Erythromycin; KAN, Kanamycin; CHL, Chloramphenicol; STR, Streptomycin; RIF, Rifampin; IMI, Imipenem; MOX, Moxifloxacin; CLO, Clarithromycin; IMI, Imipenem; STR, Streptomycin.

^a^MRSA, Methicillin-resistant *Staphylococcus aureus*.

^b^MRSP, Methicillin-resistant *Staphylococcus pseudintermedius*.

^*^References in the brackets correspond to studies that have reported resistance to the antimicrobials.

#### 3.6.2. Antimicrobial genes

Among *Staphylococcus* species, *mec*A was reported in five MRSA studies (20, 35, 36, 40, 49) and two MRSP studies (9, 42). β-lactamase gene (*bla*_CMY − 2_ gene) was reported in *Salmonella* spp. (47) and *E. coli* isolates (17, 31, 48). While the vancomycin-resistant gene (*van*A gene) was reported by one *E. faecium* study (42). The *flo* gene was identified in one *E. coli* study (31) (Table 6).

**Table 6:** The antimicrobial resistant genes isolated from bacteria associated with hospital-acquired infections data published between 2000 and 2020.

**Pathogens**	***mec*A**	***bla*_CMY − 2_**	***flo***	***van*A**
^a^MRSA	(32, 35, 36, 40, 49)^*^			
^b^MRSP	(9, 42)			
*E. coli*		(17, 31, 48)	(31)	
*E. faecium*				(42)
*Salmonella* spp.		(47)		

^a^MRSA, Methicillin-resistant *Staphylococcus aureus*.

^b^MRSP, Methicillin-resistant *Staphylococcus pseudintermedius*.

^*^References in the brackets correspond to studies that have reported resistance to the antimicrobials.

### 3.7. Zoonotic diseases

Six (22%) studies (20, 34, 35, 40, 45, 50) reported organisms associated with HAIs that are zoonotic in nature. For example, MRSA with a SCC*mec* type IV isolated in humans (28) has also been isolated in hospitalized horses (45) and hospitalized dogs (40). Similarly, three studies reported clonal MRSA lineage in animals similar to that previously reported in humans (34, 35, 40). The plasmid DH108/30218 from *E. coli* isolates which is similar to a cassette (18-ESBL 188) reported in humans (50) has been identified.

## 4. Discussion

Hospital-acquired infections and zoonosis are increasingly becoming a global concern (53). In addition, there is an increasing prevalence of resistance among these organisms to commonly used antimicrobials. Most studies that have investigated HAIs and their antimicrobial resistance profiles are in human medicine. In view of this, studies on the occurrence and resistant profile of organisms associated with hospital-acquired and zoonotic infections in veterinary medicine are needed. In this study, bacterial organisms associated with hospital-acquired and zoonotic infections isolated were identified. Furthermore, most of the organisms identified were multidrug-resistant or harbored resistant genes. Several sources of bacterial organisms associated with HAIs including HCWs, commonly used instruments, fomites, and contaminated hospital environments were also identified.

### 4.1. Hospital-acquired bacterial infections

Bacteria associated with HAIs identified MRSA, MRSP, *Enterococcus* spp., *A. baumannii, P. aeruginosa, C. difficile, E. coli*, and *Salmonella* spp., (3, 17, 20, 29, 30). The presence of these bacterial pathogens within veterinary settings is a public health concern and emphasizes the need for the implementation of infection prevention and control measures to eliminate these pathogens. The patient microbiota, healthcare workers, fomites, and the hospital environment were identified as possible sources of organisms associated with HAIs. Therefore, control measures being implemented should be source-specific and moment-specific during patient care (54).

#### 4.1.1. Sources of bacterial organisms associated with hospital acquired infections

Identification of sources of organisms associated with HAIs in veterinary settings is critical to reducing the risk of transmission to patients and humans. Therefore, it is not surprising that most studies have largely focused on the hospital environment and commonly used instruments as potential reservoirs for organisms associated with HAIs (29, 32, 39, 41, 44). Furthermore, there are ongoing epidemiological studies to understand the relationship between environmental cleanliness and the risk of transmission of HAIs in veterinary settings (4).

The intensive care unit (ICU), surgical ward, in-house laboratory, and consultation rooms were the most important environmental sources of bacteria associated with HAIs in veterinary hospitals (17, 20, 30, 36, 42). Furthermore, environmental surfaces with human contact tend to have higher contamination levels compared to those without human contact (17, 20, 40, 42), suggesting that humans may play a major role in the transmission of these organisms within the hospital environment. This is further emphasized by studies that have isolated similar pathogens strains from the environment and hands of HCWs (28, 47, 53). Therefore, HCWs in veterinary hospitals must be trained on hand hygiene compliance to reduce the risk of transmission of HAI organisms.

Fomites served as sources of HAI organisms and facilitated transmission between animal patients, the hospital environment, and humans (32). Fomites such as clippers, personnel clothing (32, 39), cell phones (41), stethoscopes (39), and weighing scales (39) were reported to be contaminated with bacteria associated with HAIs. Therefore, the development and implementation of cleaning and disinfection protocols to prevent transmission is needed (2). In addition, all surgical materials, instruments, and other fomites which increase the possibility of transmission of these organisms must be sterilized before use (36).

#### 4.1.2. Methicillin-resistant *Staphylococcus aureus*

Methicillin-resistant *Staphylococcus aureus* was among the most common organism associated with HAIs in this study (30, 37). The organisms were reported in wound infection (19, 35), invasive procedures (19, 35), skin infections (34), asymptomatic animals (45), septic arthritis, pneumonia, incisional site infection, and rhinitis (19). Studies done in veterinary medicine also reported *Staphylococcus* strains similar to those reported in humans were reported in this study (19, 27, 45). For example, Loeffler et al. (27) in the UK identified MRSA clones (CC22 and CC30) among humans working with or in close proximity to animals suggesting transmission between animals and humans is precise (37).

Unhygienic environmental conditions are a major source of MRSA (20, 40, 44). Therefore, implementing effective infection prevention and control (9, 30, 36, 37, 40, 52) and screening animals before hospitalization will reduce the spread of MRSA in veterinary hospitals. This is likely to reduce costs associated with increased length of hospital stay (19, 20, 27, 36, 45).

Most MRSA isolates in this study were resistant to β-lactam, 2nd generation cephalosporins, lincosamides, and aminoglycosides. While one study reported intermediate susceptibility to vancomycin among MRSA isolates (36). The presence of vancomycin resistance is concerning as it is the last resort for the treatment of MRSA in humans. Similarly, the presence of β-lactam resistance among staphylococci facilitated by the *mec*A gene (20, 35–37, 40, 49) is likely to contribute to resistance to other antimicrobials with a β-lactam ring (9, 40, 42, 45). Therefore, the implementation and constant review of infection control protocols are needed to help reduce the risk of the transfer of resistance genes to other organisms (9, 55–57). Without these interventions, patient care and treatment will likely be negatively impacted (36, 40).

#### 4.1.3. Methicillin-resistant *Staphylococcus pseudintermedius*

Methicillin-resistant *Staphylococcus pseudintermedius* like MRSA has emerged as a leading cause of opportunistic infections in companion animals (32, 42). The organism has been reported in asymptomatic animals, implant-associated surgical sites (41), fomites (9, 32, 41), and in the environment within the veterinary hospital (44). Therefore, colonized, and contaminated areas remain potential sources of hospital-acquired infections (32).

Areas in the veterinary facilities have been shown to harbor MRSP. These include surfaces such as tables, chairs, floors, and surgical environments (58–60). Moreover, some MRSP organisms are able to survive cleaning and disinfection (58, 59). In view of the potential resistance to disinfectants coupled with ineffective cleaning, these areas can become a source of infection for susceptible animals. Notwithstanding, some disinfectants if used at the correct concentrations are effective against MRSP (61, 62).

Of concern is that MRSP is highly resistant to antimicrobials commonly used for the treatment of *S. pseudintermedius* infections (63–65). These organisms have been isolated from the environment and hands of HCWs (42), which is concerning as it limits treatment options. Similar to MRSA, MRSP can acquire the *mec*A gene (42). Shoen et al. (9) showed coagulase positive *S. pseudintermedius* commonly isolated from the skin of dogs can acquire the *mec*A gene from a coagulase-negative *S. epidermidis* commonly found in humans.

The zoonotic cases associated with MRSP are not common (32). However, an MRSA spa type 18/t338 from animal-related fomites has been reported in humans (41). The rise in the number of MRSP cases between dogs, pet owners, and veterinary staff is concerning, therefore, effective hand hygiene should be performed before and after contact with the patient, as well as after contact with potentially contaminated environmental sites within veterinary hospitals.

#### 4.1.4. *Enterococcus* species

*Enterococcus* species are commensal of the gut flora of cats and dogs (3, 43). However, they are also opportunistic pathogens (3). In recent years, *Enterococcus* species have emerged as causes of HAIs in veterinary medicine associated with urinary tract infections (UTIs) (66). The transmission is mainly due to fecal contaminated fomites or environmental surfaces (29). These organisms can survive in a hospital environment for a long period. Furthermore, they can survive high temperatures and disinfectants such as chlorine and alcohol (42).

*Enterococcus faecium* and *E. faecalis* are the most predominant species reported in dogs (30, 43), hospital environments and in hands of HCWs (42). Of the two species, *E. faecalis* is the predominant enterococci. Multidrug-resistant enterococci have also been reported as a commensal and pathogenic organism (3, 42, 43). The presence of MDR among *Enterococcus* species has largely been attributed to overuse and misuse of antimicrobials (42, 43). It is also possible that some may have acquired resistance through other mechanisms including genetic transfer or mutation (43). For example, resistance to erythromycin has been associated with the methylation of the ribosomal target site of these antibiotics (42, 67). Nonetheless, the presence of MDR enterococci is likely to impact patient care in veterinary hospitals (42).

Of concern is the emergence of vancomycin-resistant *E. faecium* (42) which is an important antimicrobial in the treatment of enterococci infections (43, 67) and is mediated by the presence of *van*A genes. These genes are important as they confer multidrug resistance and may be transmitted to other bacterial species such as *Staphylococcus* and create even bigger problems in the treatment of HAIs (42). Furthermore, these genes can also be transferred from animals to humans (3, 43).

#### 4.1.5. *Clostridioides difficile*

*Clostridioides difficile* is found in the hospital environmental and it is difficult to eradicate (17). Both humans and animals are asymptomatically carriers of the organism. In humans, its presence has been attributed to the overuse of antimicrobials. However, in veterinary medicine there is limited information about the organism. Therefore, future studies should look at whether the overuse of antimicrobials could be a driver of *C. difficile* in veterinary settings (17). The ability of the pathogen to survive harsh environmental conditions and resistance to most disinfectants makes it a suitable indicator of the effective IPC measures (17). Therefore, it is possible that this organism can also be used as an indicator of effective infection prevention and control in veterinary hospitals.

#### 4.1.6. *Acinetobacter baumannii*

*Acinetobacter baumannii* causes life-threatening infections in both humans and animals. This organism has been reported in UTIs, pyothorax, upper airway obstruction, bloodstream infection, and wound infections in animals (39). In infected animals, it is associated with increased morbidity and prolonged length of hospital stay (68). *Acinetobacter baumannii* survives on dry surfaces (29, 39, 69). Therefore, commonly used fomites, bed rails, cages, and examination tables could serve as reservoirs for *A. baumannii*.

The organism can survive stressful environmental conditions and remains viable on different surfaces (70). However, if used at correct concentrations, sodium hypochlorite (bleach) and 70% ethanol are effective against *A. baumannii* (70, 71). Lanjri et al. (71) observed that chlorhexidine digluconate was effective against *A. baumannii*. La Forgia et al. (72) also reported that sodium hypochlorite was effective in reducing the incidence rate of *A. baumannii* in hospital settings. In light of this findings, choosing the correct disinfectant is important in reducing cases of *A. baumannii* in hospital settings.

Most *A. baumannii* are multiple drug resistant with a high prevalence of resistance toward cephalexin, enrofloxacin, amoxicillin-clavulanic acid, sulphamethoxazole-trimethoprim, and tetracycline (39). Resistance to the above antimicrobials is concerning as these antimicrobials are commonly used for the treatment of bacterial infections in small animal medicine (68). In addition, the *bla*OXA-51 gene reported in an *A. baumannii* isolate from pigs has also been reported in humans (67).

#### 4.1.7. *Escherichia coli*

*Escherichia coli* is commonly reported in UTIs and bloodstream infections (17, 31, 39, 73). The bacterium spreads from patient to patient *via* fecal contaminated hands of HCWs and shared equipment (31). Given, that environmental surfaces could potentially be a reservoir of *E. coli*, measures to minimize fecal contamination in companion animal hospitals including cleaning and disinfection of the hospital environment should be implemented. Moreover, Sanchez et al. (31) shows the transfer of *E. coli* isolates with similar antimicrobial resistance patterns between two different animals admitted to the same ICU.

In this study, *E. coli* isolates exhibited resistance toward cephalosporins and β-lactams including amoxycillin-clavulanic acid. This broad-spectrum antimicrobial resistance among *E. coli* is attributed to the presence of *amp*C like gene, *bla*_CMY2_ (17, 31), which has been identified to be of public health concern (17). Another study reported resistance among *E. coli* isolates to chloramphenicol mainly due to the presence of *cml*A homologue *flo* among gram-negative bacteria (31). The presence of these genes has also been linked to the development of resistance to other commonly used antibiotics such as gentamycin, spectinomycin, and sulfadimethoxine (31, 39, 50). Considering this resistance, strict guidelines should be implemented on the prudent use of antimicrobials in veterinary medicine.

#### 4.1.8. *Salmonella* species

Although most animals are asymptomatic carriers of *Salmonella* spp., they shed the bacterium in high quantities through their feces resulting in *Salmonella* outbreaks in equine veterinary hospitals (46, 47). Furthermore, infections associated with *Salmonella* species have also been reported in bovine with diarrhea, fever, dehydration (38) and colic in horses (47). In affected animals, the disease is characterized by high morbidity and mortality. There is a potential spread of organisms and the occurrence of zoonotic infection that may result in the closure of facilities and a loss of income for the hospital (38, 46). Therefore, personnel working in close contact with infected animals are at an increased risk of infection (46).

Managing transmission in the veterinary settings remains a challenge as *Salmonella* can persist in the environment for a long time. Rodents and contaminated feed could also be a source (46, 47). Therefore, biosecurity measures must be intensified in veterinary hospitals to reduce the risk of transmission. Additionally, education programs can also be developed targeting specific aspects of hygiene, movement control, and cleanliness of equipment.

*Salmonella* isolates were resistant to ceftiofur, gentamycin, amoxicillin, ampicillin, streptomycin, and trimethoprim/ sulfadiazine (46, 47). One study reported the presence of the cephalomycinase gene, *bla*_cmy2_ (47) which has been associated with cephalosporin resistance among *Salmonella* species. This gene has also been reported to mediate resistance to amoxicillin, amoxicillin-clavulanic acid, cephalothin, cefoxitin, ceftiofur, and ceftriaxone (47).

## 5. Conclusion

Organisms associated with hospital-acquired and zoonotic diseases were reported from clinical cases, environmental surfaces, and items used in veterinary service. The hospital environment with human contact was the most reported source of organisms associated with HAIs. These results suggest that humans play a crucial role in the transmission of HAIs in veterinary hospitals.

Among the organisms reported, MRSA *and* MRSP were the most reported HAI organisms in veterinary facilities. Other organisms identified include *E. coli, C. difficile, A. baumannii, Salmonella* spp., and *Enterococcus* species. Some of these isolates reported in veterinary settings share similar clonal lineage to those reported in humans. Some organisms exhibit a high prevalence of antimicrobial resistance and contain genes known to be associated with antibiotic resistance.

These results suggest that strict infection prevention and control practices must be in place, monitored and modified when necessary to curb the occurrence and transmission of organisms associated with HAIs in veterinary hospitals. In addition, continuous surveillance of HAI organisms and their antimicrobial resistance patterns in veterinary hospitals should be emphasized. Further research needs to be done on *C. difficile* as a potential indicator of effective infection prevention and control practices in veterinary facilities.

## Data availability statement

The original contributions presented in the study are included in the article/Supplementary material, further inquiries can be directed to the corresponding authors.

## Author contributions

DS was involved in study design, data analysis, interpretation of results, and writing of manuscript as well as extensive editing of the manuscript. DQ was involved in study design, data management, analysis, and interpretation as well as reviewing of the manuscript draft. JO and MK were involved in study design, data analysis, and interpretation as well as editing of the manuscript. All authors have read and approved the final manuscript.
