# Supplementary material for: Hospital-acquired and zoonotic bacteria from a veterinary hospital and their associated antimicrobial-susceptibility profiles: A systematic review
Source: Front Vet Sci. 2023 Jan 9;9:1087052. doi: 10.3389/fvets.2022.1087052 (PMC9868922; doi:10.3389/fvets.2022.1087052)
Supplement: Supplementary file 1 [file Table_1.DOCX]

**Annexure A: Risk of bias of the reported articles using the STROBE‐Vet Checklist**

|  | **Items on STROBE‐Vet Checklist (Sargeant et al., 2016)** | | | | | | | | | | | | | | | | | | | | | | |
| --- | --- | --- | --- | --- | --- | --- | --- | --- | --- | --- | --- | --- | --- | --- | --- | --- | --- | --- | --- | --- | --- | --- | --- |
|  | **1** | | **2** | **3** | **4** | **5** | **6** | **7** | **8** | **10** | **11** | **12** | **13** | **14** | **15** | **16** | **17** | **18** | **19** | **20** | **21** | **22** | |
| **Article authors** | **Title** | **Abstract** | **Background/**  **Rationale** | **Objectives** | **Study design** | **Settings** | **Participants** | **Variables** | **Data Sources** | **Bias** | **Study size** | **Statistical methods** | **Descriptive data** | **Outcome data** | **Main results** | **Other analysis** | **Key results** | **Strength and Limitations** | **Interpretation** | **Generalizability** | **Limitations** | **Other** |  |
|  |  |  |  |  |  |  |  |  |  |  |  |  |  |  |  |  |  |  |  |  |  | **F** | **RA** |
| Murphy et.al 2010 |  |  |  |  |  |  |  |  |  |  |  |  |  |  |  |  |  |  |  |  |  |  |  |
| Mount et.al 2016 |  |  |  |  |  |  |  |  |  |  |  |  |  |  |  |  |  |  |  |  |  |  |  |
| Boerlin et.al 2001 |  |  |  |  |  |  |  |  |  |  |  |  |  |  |  |  |  |  |  |  |  |  |  |
| Aksoy et.al 2010 |  |  |  |  |  |  |  |  |  |  |  |  |  |  |  |  |  |  |  |  |  |  |  |
| Stella et.al 2020 |  |  |  |  |  |  |  |  |  |  |  |  |  |  |  |  |  |  |  |  |  |  |  |
| Heller et.al 2009 |  |  |  |  |  |  |  |  |  |  |  |  |  |  |  |  |  |  |  |  |  |  |  |
| Cummings et.al 2010 |  |  |  |  |  |  |  |  |  |  |  |  |  |  |  |  |  |  |  |  |  |  |  |
| Ng et al 2016 |  |  |  |  |  |  |  |  |  |  |  |  |  |  |  |  |  |  |  |  |  |  |  |
| Rojas et.al 2017 |  |  |  |  |  |  |  |  |  |  |  |  |  |  |  |  |  |  |  |  |  |  |  |
| Weese, Caldwell et al 2006 |  |  |  |  |  |  |  |  |  |  |  |  |  |  |  |  |  |  |  |  |  |  |  |
| Julian et al 2012 |  |  |  |  |  |  |  |  |  |  |  |  |  |  |  |  |  |  |  |  |  |  |  |
| Ossiprandi et al 2008 |  |  |  |  |  |  |  |  |  |  |  |  |  |  |  |  |  |  |  |  |  |  |  |
| Lutz et al 2013 |  |  |  |  |  |  |  |  |  |  |  |  |  |  |  |  |  |  |  |  |  |  |  |
| Shoen et.al 2019 |  |  |  |  |  |  |  |  |  |  |  |  |  |  |  |  |  |  |  |  |  |  |  |
| Hoet et al 2011 |  |  |  |  |  |  |  |  |  |  |  |  |  |  |  |  |  |  |  |  |  |  |  |
| Van den Eede et al 2009 |  |  |  |  |  |  |  |  |  |  |  |  |  |  |  |  |  |  |  |  |  |  |  |
| Loeffler et al 2010 |  |  |  |  |  |  |  |  |  |  |  |  |  |  |  |  |  |  |  |  |  |  |  |
| Soza-Ossandon et al 2020 |  |  |  |  |  |  |  |  |  |  |  |  |  |  |  |  |  |  |  |  |  |  |  |
| Ward et al 2005 |  |  |  |  |  |  |  |  |  |  |  |  |  |  |  |  |  |  |  |  |  |  |  |
| Sidjabat et al 2009 |  |  |  |  |  |  |  |  |  |  |  |  |  |  |  |  |  |  |  |  |  |  |  |
| Sanchez et al 2002 |  |  |  |  |  |  |  |  |  |  |  |  |  |  |  |  |  |  |  |  |  |  |  |
| Baek et al 2018 |  |  |  |  |  |  |  |  |  |  |  |  |  |  |  |  |  |  |  |  |  |  |  |
| Pailhories et al 2015 |  |  |  |  |  |  |  |  |  |  |  |  |  |  |  |  |  |  |  |  |  |  |  |
| Singh et al 2013 |  |  |  |  |  |  |  |  |  |  |  |  |  |  |  |  |  |  |  |  |  |  |  |
| Weese et.al 2006 |  |  |  |  |  |  |  |  |  |  |  |  |  |  |  |  |  |  |  |  |  |  |  |
| Van Duijkeren et al 2004 |  |  |  |  |  |  |  |  |  |  |  |  |  |  |  |  |  |  |  |  |  |  |  |
| Bagcil et al 2006 |  |  |  |  |  |  |  |  |  |  |  |  |  |  |  |  |  |  |  |  |  |  |  |
|  |  |  |  |  |  |  |  |  |  |  |  |  |  |  |  |  |  |  |  |  |  |  |  |
| F =Funding | RA= Role of authors | | | | | | | | | |  |  |  |  |  |  |  |  |  |  |  |  |  |
| Shading interpretations: | White = Fully attained requirements for the specified item | | | | | | | | | | | | | | | | | | | | | | |
|  | Grey = Partially attained requirements for the specified item | | | | | | | | | | | | | | | | | | | | | | |
|  | Black = Requirements for the specified item not attained | | | | | | | | | | | | | | | | | | | | | | |
